# Mitochondrial metabolism in neural stem cells and implications for neurodevelopmental and neurodegenerative diseases

**DOI:** 10.1186/s12967-024-05041-w

**Published:** 2024-03-04

**Authors:** C. Garone, F. De Giorgio, S. Carli

**Affiliations:** 1 Department of Medical and Surgical Sciences, Alma Mater Studiorum-University of Bologna, Bologna, Italy; 2 IRCCS Istituto Delle Scienze Neurologiche di Bologna, UO Neuropsichiatria Dell’età Pediatrica, Bologna, Italy

**Keywords:** Mitochondrial metabolism, Neurodegenerative disorders, Neuronal development, Stem cells, Wnt, HIF-1A, mTOR

## Abstract

Mitochondria are cytoplasmic organelles having a fundamental role in the regulation of neural stem cell (NSC) fate during neural development and maintenance.

During embryonic and adult neurogenesis, NSCs undergo a metabolic switch from glycolytic to oxidative phosphorylation with a rise in mitochondrial DNA (mtDNA) content, changes in mitochondria shape and size, and a physiological augmentation of mitochondrial reactive oxygen species which together drive NSCs to proliferate and differentiate. Genetic and epigenetic modifications of proteins involved in cellular differentiation (Mechanistic Target of Rapamycin), proliferation (Wingless-type), and hypoxia (Mitogen-activated protein kinase)–and all connected by the common key regulatory factor Hypoxia Inducible Factor-1A–are deemed to be responsible for the metabolic shift and, consequently, NSC fate in physiological and pathological conditions.

Both primary mitochondrial dysfunction due to mutations in nuclear DNA or mtDNA or secondary mitochondrial dysfunction in oxidative phosphorylation (OXPHOS) metabolism, mitochondrial dynamics, and organelle interplay pathways can contribute to the development of neurodevelopmental or progressive neurodegenerative disorders.

This review analyses the physiology and pathology of neural development starting from the available in vitro and in vivo models and highlights the current knowledge concerning key mitochondrial pathways involved in this process.

**Supplementary Information:**

The online version contains supplementary material available at 10.1186/s12967-024-05041-w.

## Introduction

Mitochondria are organelles regulating a plethora of cellular processes, although their main role is to convert nutrients into energy through oxidative phosphorylation (OXPHOS) in which pyruvate from the glycolytic pathway enters the mitochondria and is converted into acetyl-CoA, whereupon it enters the Tricarboxylic Acid (TCA) cycle. Nicotinamide adenine dinucleotide (NADH) and Flavin adenine dinucleotide (FADH) synthesized in the TCA then generate an electrochemical gradient within the Electron Transport Chain (ETC, Complexes I–IV) that reaches Complex V (ATP synthase), generating ATP [1].

A unique characteristic of mitochondria is the presence of mitochondrial DNA (mtDNA) that in humans is a circular molecule of 16.5 kb including 37 genes encoding 2 rRNA and 22 tRNA for the translation of mitochondrial proteins and 13 OXPHOS complex subunits [2]. mtDNA copies increase during the organism's development through a process regulated by methylation of the gene encoding the catalytic subunit of polymerase γ, establishing a set point for differentiation into different cell types and varying between 200 to 10,000 copies in somatic cells across different tissues [1]. Importantly, mitochondrial structure and function are tightly regulated through the expression of proteins under the dual control of mitochondrial and nuclear genes [3]. At the morphological level, mitochondria have a double-membrane configuration, with inner and outer membranes (IMM and OMM, respectively), and intermembrane space, all involved in coordinated cycles of fission and fusion that determine the length and morphology of mitochondria within different cell populations [4, 5]. The fusion process is mediated by Mitofusin 1 (MFN1) and Mitofusin 2 (MFN2), forming an oligomeric complex on the OMM, and by the Optic atrophy protein (Opa1) which mediates the IMM fusion and promotes the cristae remodeling [6, 7]. Mitochondrial fission requires the Dynamin-related protein 1 (DRP1) that is selectively recruited to the endoplasmic reticulum (ER)-mitochondria interface to regulate the formation of constriction sites. ER tubules circumscribe mitochondrial regions destined for removal and the actin cytoskeleton with the assembly of Drp1 at ER-mitochondria contact sites are responsible for the division of both OMM and IMM. Fis1 protein is instead responsible for the inhibition of the fusion machinery[8, 9]. The coordination of mitochondrial fusion and fission is fundamental in brain development [10–13]: mitochondria shape varies from tubular, in human pluripotent stem cells (hPSCs), to non-fused and fragmented mitochondria during the transition to proliferative neuro progenitor cells (NPCs) [14, 15]. The transition between NSCs to NPCs is ruled by a metabolic shift from glycolysis to OXPHOS [16, 17].

Different molecular pathways are involved in the self-renewal and differentiation of NSCs into mature neurons, including the Wingless-type (Wnt), mechanistic Target of Rapamycin (mTOR), and hypoxia signaling. Each of these pathways synergically influences NSCs' fate at metabolic, transcriptomic, and proteomic levels [18–20].

In this review, we will focus on the role of mitochondria in NSCs' fate in embryonic and adult neurogenesis by detailing the genetic and epigenetic regulatory mechanisms and their downstream effects on different neuronal lineages in both physiological and pathological conditions.

## Developmental and adult neurogenesis

Neurogenesis is a process precisely regulated by molecular signaling pathways that guide NSCs–in embryo as well as adult brain niches–towards differentiation into NPCs, neuronal cell precursors, and eventually neurons [21, 22].

Stem cells are distinct from other cells for their capacity for self-renewal and pluripotency [23–25] and for their ability to generate any cell type of the three primary embryonic germinal layers [26]. Cell division can be symmetric, generating two identical daughter cells, or asymmetric generating one identical daughter cell and one precursor with a different fate [27, 28].

Stem cells of the nervous system, named neuroepithelial (NE) cells, are polarized cells with epithelial traits including the presence of tight/adherent junctions [29]. During neural development, NE cells undergo several cycles of symmetric and asymmetric divisions to maintain the stem cell pool and generate radial glial (RG) cells [30]. RG are fate-defined and CNS-specific cells showing some astroglia features such as the glial fibrillary acidic protein (GFAP), the astrocyte-specific glutamate transporter (GLAST), and vimentin [31, 32]. RG cells in the ventricular zone (VZ) follow asymmetric division generating identical daughter cells and basal progenitors (BP), also known as intermediate progenitor (IP) cells, which subsequently migrate to the subventricular zone (SVZ) where they divide symmetrically into neuronal cells, assemble the neocortex and form the cortical layer, with younger neurons at the outer layers [33, 34]. Then, neurons can migrate in two different ways: radial and tangential migration; the first one represents the principal form of migration in the cerebrum, in which neurons move perpendicularly to the ventricular surface since the proper layer; the second is mainly used by inhibitory interneurons from the ganglionic eminences to migrate in a tangential way through the cortical plate to finally extend their axons with the proper target [35]. Interestingly, new-born neurons show small mitochondria, thus suggesting an intensification of mitochondrial fission throughout the neurogenic transition, whereas mitochondria size increases during neuronal maturation [36, 37] Importantly, the migration and differentiation of BP cells occur mainly during embryonic development although it is partially maintained in postnatal brain niches located in the subgranular zone (SGZ), within the dentate gyrus of the hippocampus, and in the SVZ, which supplies cells to the olfactory bulb (OB) through the rostral migratory stream (RMS) [38, 39]. BP cells in the SVZ are thought to be key players for neocortical expansion, especially in those species with thicker neocortices [40–42]. After neuron formation RG cells may proliferate to generate oligodendrocytes and astrocytes.

In the adult mammalian brain, neurogenesis endures in two specific neurogenic niches: the SVZ, which provides neurons for the striatum, and the subgranular zone of the dentate gyrus (DG) [22] (Urbán and Guillemot, 2014). Importantly, DG is developed from distinct progenitor cells, the dentate neuroepithelium (DNE), that exclusively generate granule neurons in this brain region. Contrary to the high proliferative rate of embryonic NSCs, adult NSCs persist for long periods in the G0 of the cell cycle to preserve tissue homeostasis and prevent stem cell exhaustion [43, 44]. Adult neurogenic niches are constituted of different types of cells, including neuronal progenitor cells, glial cells (astrocytes and NG2 glia), and ependymal cells [45]. Astrocytes provide GFAP-positive NSCs and promote cell proliferation and differentiation. NG2-expressing glial cells generate myelinating oligodendrocytes and share synapsis together with neurons. Ependymal cells–found in the SVZ and central canal of the spinal cord– provide a multiciliate structure surrounding the NSCs’ apical processes [46–51].

## Mitochondria in NSCs fate decision

### Mitochondrial re-modeling and NSCs differentiation

**Fig. 1:**
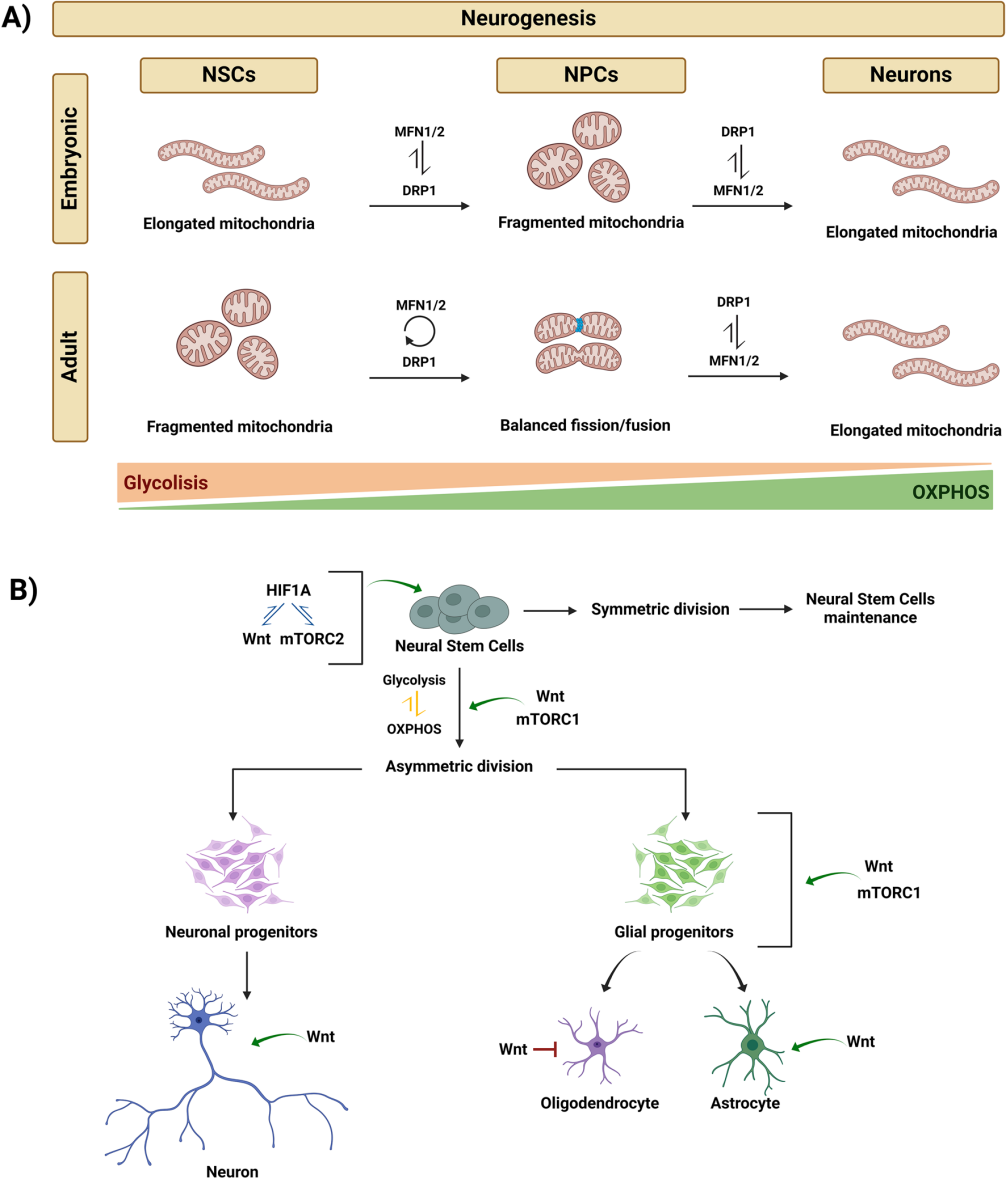
Mitochondrial regulation of embryonic neurogenesis is modulated by the synergistic action of HIF1α, mTOR, and Wnt–(**a**) During embryonic and adult neurogenesis, changes in the metabolism of differentiating cells are accompanied by rearrangements of mitochondrial morphology. **b** NSCs' commitment to differentiation starts with a metabolic switch from glycolysis to OXPHOS inducing stem cells' asymmetric division and generating neuronal and glial progenitors. Differentiation may further proceed to neurons or oligodendrocytes/astrocytes. HIF1α together with the mTOR and the Wnt signaling are key modulators of these steps (Created with Biorender)

Changes in mitochondria morphology are hallmarks of the differentiated and pluripotent stages of different subtypes of neuronal and glial cells in both embryonic and adult neurogenesis. Mitochondria-to-nuclear retrograde signaling regulates the transcription of genes responsible for differentiation. This signaling determines mitochondrial dynamics changes fundamentally in committing NSCs to differentiation [16]. Drp1 (fission) regulates the cycles of mitochondria re-modeling, inducing mitochondrial fragmentation to promote glycolytic metabolism while Mfn1/2 are fundamental for mitochondrial fusion to promote OXPHOS metabolism [52] (Fig. 1).

A recent study investigated the re-modeling of mitochondria during NSCs conversion into neurons, in mouse RGCs and in human NPCs derived from induced pluripotent stem cells (iPSCs). During mitosis, undifferentiated cells following the self-renewal path show fused mitochondria while NSCs committed to becoming neurons have fragmented mitochondria [37]. Moreover, it has been demonstrated the presence of a time window of three hours after mitosis in mouse cells and of six hours in human cells during which these mechanisms of mitochondrial re-modeling can be modulated or even reversed [37]. Changes in mitochondrial dynamics might therefore precede the metabolic switch necessary for differentiation initiation.

Moreover, PSCs, as well as quiescent stem cells within adult niches, rely on two anabolic pathways for cell-cycle progression: the glycolytic pathway terminating with the conversion of pyruvate to lactate and the glutaminolysis responsible to fuel the TCA cycle with glutamate [53]. Glycolysis is less efficient in terms of energy intake (only two ATP molecules per glucose unit) than OXPHOS and the TCA cycle. However, it is faster than oxidative phosphorylation, can be oxygen-independent, and provides intermediate metabolites fundamental for amino acids, lipids, and nucleotide synthesis. It can thus support stem cells in maintaining both proliferation and pluripotency [54, 55]. By avoiding mitochondrial oxidative metabolism, cells relying on glycolysis also produce less reactive oxygen species (ROS) and, consequently, may potentially cause less damage to both nDNA and mtDNA [56]. To maintain the high glycolytic rate in ESCs, the activity of HK2, an isoform of hexokinase (HK), and PKM2, an isoform of pyruvate kinase (PK), increase through a mechanism regulated by the octamer-binding transcription *factor* 4 (OCT4), a key transcription factor involved in stem cell pluripotency [52, 57, 58]. SIRT1 and SIRT2–NAD-dependent deacetylases–are upregulated and downregulated respectively through an OCT4-dependent mechanism to induce hypoacetylation of glycolytic enzymes and to accelerate glycolysis [59]. Moreover, GLUT1, which plays a key role in maintaining glucose uptake and consequently glycolysis, is highly expressed in pluripotent stem cells and its novel identified enhancer element, GE, is activated by the binding of SOX2, OCT4, and NANOG [60]. In addition, the increased Myc expression, regulated by the miRNAs miR-290/371 and the Methyl-CpG Binding Domain Protein 2 (Mbd2), also augments the expression of both pyruvate kinase isozymes M2 (PKM2) and Lactate Dehydrogenase A (LDHA) enhancing glycolysis [61].

Glutaminolysis is a metabolic pathway that fuels the TCA cycle independently by glycolysis. It converts glutamine to glutamate and a-ketoglutarate (aKG), thus allowing the TCA cycle to be converted into a “three-quarter cycle” metabolic pathway that starts at the aKG step and stops at the oxaloacetate step, which is a primary metabolic source for anabolic processes, such as the synthesis of nucleotides and various amino acids necessary for cell progression [62].

In vitro and in vivo studies have demonstrated that both pathways play important roles in NPC proliferation in the developing cortex. Murine genetic manipulation of glycolysis with ablation of apoptosis-inducing factor, an essential mitochondrial protein for the activity of complex I of the respiratory chain, increases glycolysis and NPCs proliferation and impairs the generation of neurons from the NPCs causing a thinner neocortex [63]. Similarly, murine proteins involved in brain size determination such as Mcph1 and ARHGAP11B are localized in the mitochondria and play their primary role in stimulating aRG and BP proliferation, respectively, via glutaminolysis [62, 64, 65].

Epigenetic and biochemical signal interplay regulates the expression of metabolic enzymes [16] essential for switching between glycolysis and OXPHOS, with mitochondrial pathways such as the TCA cycles and the one-carbon metabolism playing a pivotal role in the epigenetic modifications, and HIF-1α pathways influencing downstream effects on mitochondrial function [66].

At epigenetic levels, the methionine pathway of 1C metabolism is implicated in NSCs self-renewal and differentiation by regulating the methylated state of specific genetic targets via the generation of a methyl donor, the S-adenosyl methionine (SAM) [67]. Specifically, pluripotency depends on histone H3 lysine 4 trimethylation (H3K4me3) and histone H3 lysine 27 trimethylation (H3K27me3), themselves regulated by SAM levels [68–70]. One-carbon metabolism deficiency inhibits the proliferation of both embryonic NSCs in vitro and adult hippocampal NSCs in vivo [71, 72], whereas fueling the 1C metabolism with in vitro folate supplementation increases NSCs proliferation and neuronal differentiation by enhancing DNA methyltransferases [66, 73, 74].

Two metabolites essential for epigenetic modifications in the TCA cycle are αKG and acetyl-CoA, respectively modulating histone methylation and acetylation [75]. An αKG-dependent mechanism also modulates the repressive histone modifications of H3K0me3 and H3K27me3, while acetyl-CoA regulates H3K27 acetylation [76–78]. In addition, high α-KG levels prevent the accumulation of repressive histone modifications and DNA methylation and stimulate pluripotency in mouse embryonic stem cells, since α-KG is a substrate of Jumonji-C domain-containing histone demethylases (JMJC) and Tet-DNA demethylases [66].

Deficiency of key enzymes modulating α-KG and acetyl-CoA levels results in neocortical malformations. One among the others, mutations encoding a mitochondrial thiamine pyrophosphate carrier in the SLC25A19 gene cause a reduction of thiamine, a coenzyme of the a-ketoglutarate dehydrogenase (KGDH) complex, and consequently, of decreased KGDH activity. This defect affects neuronal proliferation and is responsible for the Amish lethal microcephaly [62] Additional file 1.

In adult neurogenesis, NSC and NP cells are also characterized by a specific epigenetic signature in lipid metabolism. Adult NSC and NP cells necessitate Fasn-dependent lipogenesis for proliferation, and it was found that Spot14 downregulates NSC and NP cells proliferation by reducing the availability of malonyl-CoA6, an essential substrate for Fasn to fuel lipogenesis, and thus de novo lipid synthesis [78]. In vitro and in vivo models have demonstrated an increased de novo lipogenesis through fatty acids synthase in proliferating NP and NSC cells and a reduction in quiescent cells. Lipids seem important not only as building blocks for membranes but also as an alternative energy source to glucose through fatty acid oxidation [79, 80].

### HIF1a-related pathway plays a central role in NSC differentiation

The expansion of aRG during the early phase of neocortical neurogenesis presumably occurs under hypoxic conditions. Hypoxia-inducible-factor HIF-1α (HIF-1α) expression in aRG [81, 82] and glycogen accumulation in aRG [83] both support this notion.

HIF-1 is a transcription factor of the hypoxia signaling pathway organized in two subunits (α and β) that plays a pivotal role in regulating the expression of several glycolytic genes, including HK and PK [52, 84]. In fact, the metabolic trait of PSCs, which have lower cytochrome C oxidase (COX) expression, is similar to the one observed in cancer cells, called the Warburg effect, which is favored by HIF-1 [85, 86].

HIF1α is also involved in the transition from OXPHOS to aerobic glycolysis in the reprogramming of somatic cells into iPSCs: during early stages of the reprogramming, the metabolic shift starts with the so-called OXPHOS burst, a mechanism induced by c-Myc dependent upregulation of the peroxisome-proliferator-activated receptor-gamma co-activator-1 α/β (PGC-1α/β) and the estrogen-related nuclear receptors (ERRα/γ), and regulated by the nuclear factor erythroid 2-related factor 2 (NRF2) which activates HIF1α allowing expression of glycolytic genes [87–90].

The HIF1α factor is a central sensor for other two important pathways controlling neurogenesis – Wnts and mTOR – whose actions are detailed in the following sections. mTOR signaling regulates HIF1α stability and transcriptional activity by phosphorylating Mint3, a member of the X11 protein family, and by the direct interaction between mTORC1 subunit Raptor and the mTOR signaling motif of HIF1α [91, 92]. Therefore, mTORC1 increases glucose uptake and glycolysis through the induction of HIF1α [93]. Additionally, hypoxia also stabilizes the TSC complex (Tuberous sclerosis complex) causing inhibition of mTORC1 [93].

The very beginning of the NSC commitment to differentiate into neurons is characterized by a metabolic switch from aerobic glycolysis to OXPHOS. This begins with lactate dehydrogenase (LDH) downregulation followed by the shift of PK from PKM2 to PKM1, both involved in aerobic glycolysis. A decrease in HK2 and the transporter GLUT1/3 then reduces glucose entry into the metabolic pathway at a minimum required to generate pyruvate for the TCA cycle. Moreover, throughout the neuronal differentiation of human NPCs, a significant reduction of the uncoupling protein 2 (UCP2) has been observed, which promotes aerobic glycolysis by limiting the pyruvate entry into the TCA cycle [94–96].

On the other hand, hypoxia is able to increase HIF1α, β-catenin, and cyclin D1 levels, suggesting that HIF1α plays a role in Wnt pathway regulation both during embryonic development and neural repair [19].

### mTORC1/mTORC2 ratio regulates mitochondrial biogenesis and function and it is pivotal in cell differentiation and commitment

mTOR is a phosphoinositide 3-kinase (PI3K) family kinase that plays a key role in multiple cellular processes including protein synthesis, mitochondrial energy production, lipid and nucleotide synthesis, and autophagy to fuel cell growth, proliferation, and cell subtypecommitment [93].

mTOR forms the mTOR 1 (mTORC1) and 2 (mTORC2) complexes, which differ in their composition, regulation, and downstream targets of cellular homeostasis. Both complexes serve as central sensors of metabolic changes occurring during neurogenesis and act as regulators of mitochondrial function and biogenesis.

mTORC1 stimulates mitochondrial biogenesis by regulating the transcription and translation of mitochondrial proteins via a direct effect on the expression of nuclear-encoded mitochondrial proteins such as TFAM, mitochondrial ribosomal proteins, components of complex I and V, or by indirectly modulating proteins such as Yin Yang 1 (YY1) and peroxisome proliferator-activated receptor-gamma coactivator 1 a (PGC-1α). It also suppresses autophagy by inhibiting ULK1 (directly or via ATG13) and the nuclear translocation of transcription factor EB (TFEB). Additionally, mTORC1 regulates mitochondrial energy by modulating glycolysis through the 4E-BP1-dependent translational activation of the HIF1α and regulates glutaminolysis by inhibiting SIRT4 [93].

mTORC2 controls mitochondria-associated ER membrane (MAM) integrity and mitochondrial function [93]. Following recruitment to the MAM, mTORC2 suppresses mitochondrial ATP production, membrane potential, and calcium uptake by phosphorylating MAM resident proteins [93].

In neurogenesis, different levels of mTORC1 and mTORC2 act as regulators of NSCs differentiation, progenitor migration, neuronal differentiation, and dendritogenesis [18]. Activated by insulin growth factor-1 (IGF-1), mTORC2 phosphorylates PKB/Akt, promoting cell survival and proliferation in NSCs, while mTORC1 alone is sufficient to enhance cycle entry [97–100]. In ESCs, TSC1/TSC2 and DEP domain-containing mTOR interacting proteins (DEPTOR) maintain a level of mTORC2 higher than mTORC1, consequently, reducing the protein synthesis and cell stemness [101, 102]. In the SVZ, activation of mTORC1 promotes NSC proliferation and neural lineage expansion as well as the generation of Tbr2 + intermediate progenitors [97, 103]. The PI3K-Akt-mTOR pathway is directly involved in the development and differentiation of the cortex, as its dysfunction causes cortical malformation, aberrant migration, and neuron reductions [104–106]. mTOR signaling, in fact, regulates apical and basal dendrite formation, as deletion of both PTEN, inhibitor of mTORC2, and TSC1, an inhibitor of mTORC1, enhance dendritic branches [107–109]. mTORC1 – upregulated by glutamate signaling through mGluR and N-methyl-D-aspartate–activates the initiation of protein translation at dendritic spines, favoring synaptic plasticity [110, 111]. Impairments in mTORC1 activity can therefore cause abnormal differentiation or reduced proliferation of neural progenitor cells and can be responsible for neurodevelopmental disorders collectively referred to as mTORpathies [112, 113]. Tuberous sclerosis complex (TSC) is a typical mTORpathy due to autosomal dominant inherited variants in *Tsc1* or *Tsc2* gene. Defective TSC proteins lead to hyperactivation of mTOR and [18, 114, 115] causes three main clinical symptoms in patients: focal dysplasia, hamartomas, and brain dysfunction (epilepsy, autism spectrum disorders, and intellectual disability). *Tsc1/2* deficient mice reproduce the human disease and confirm the impairments in mitochondrial homeostasis with reduction of mitochondria and increased fragmentation [8, 116].

### The Wnt pathway regulates cellular proliferation and neuronal organization

Wnt signaling plays relevant roles in neural embryogenesis and adult neurogenesis [20]. It is characterized by 19 cysteine-rich proteins that activate different types of Wnt signaling. The three best-known signaling pathways are the canonical Wnt/β-catenin pathway and the two non-canonical planar cell polarity (PCP) and Wnt/Ca^2+^ pathways [117, 118]. In the canonical Wnt/β-catenin pathway, Wnt binds to the receptor LRP5/6 and Frizzled co-receptor activating the Dvl proteins, thus inhibiting the formation of the destruction complex, which includes GSK3α/β. β-catenin translocates then into the nucleus where it binds to the transcription factors LEF1/TCFs to activate Wnt target genes transcription [20, 119]. In the PCP pathway, binding of Wnt activates the Dvl proteins inducing microtubule re-organization through the activation of Rho-GTPases and c-Jun-N-terminal kinase (JNK), while rising intracellular Ca^2+^ with activation of protein kinase C (PKC), Ca^2+^/calmodulin-dependent protein kinase II (CamII) and nuclear factor of activated T cells (NFAT) in the Wnt/Ca2 + pathway [120–123].

The Wnt pathway plays a crucial role in stem cell maintenance, neuronal cell maturation, axon remodeling, dendritic morphogenesis, and adult tissue [124, 125], largely mediated through its regulator effect on mitochondria [124]. In fact, Wnt pathway activation influences OXPHOS activity, mitochondrial biogenesis and dynamics, and ROS production [124, 125].

Large shRNA screening has shown Wnt signaling to regulate 150 genes of mitochondrial function. Specifically, Wnt increases the expression of key mitochondrial OXPHOS genes, such as ATP synthase subunit (ATP5g1), cytochrome c (Cyc1), and mitochondrially encoded cytochrome oxidase subunit 2 (Cox2) and consequently increases OXPHOS activities through the canonical pathway involving TCF4 [124]. Mitochondrial proliferation is instead induced by a cascade of events activated by the IRS-1 adaptor protein and activate TCF4 targets such as the transcription factor Myc, a pleiotropic regulator of cellular functions, including growth, differentiation, and apoptosis, thus stimulating a cascade of gene expression responsible for mitochondrial proliferation [124].

In addition, recent evidence has demonstrated an interplay between mitochondrial fitness (energy production and dynamics) and the Wnt pathway. A decrease in mitochondrial ATP synthesis induces ER stress which, in turn, impairs β-catenin stability, ultimately reducing the expression of Wnt-related genes [126], as it was demonstrated in a mitochondrial disease model of GRACILE syndrome due to impaired complex III function [127].

In embryogenesis, Wnt proteins act as important morphogens, while in the adult brain, they contribute to NSC niche homeostasis by driving differentiation and self-renewal [128]. During embryonic development, Wnt signaling is required for correct posteriorization of the neural plate and plays a role in neuronal migration, polarization, synapse formation, and dendrite development [129, 130]. Studies in mice demonstrated that both the Wnt/PCP and the Wnt β-catenin pathways are involved in neural tube closure [131]. Moreover, in the developing neural tube, the Wnt signaling is expressed as a gradient that goes from high dorsally to low ventrally, with Wnt1 and Wnt3a being particularly important for the formation of brain structures [132]. In postnatal neural progenitors, β-catenin transcription promotes cell proliferation in physiological and ischemic conditions with expression of Wnt members observed within the mouse adult SVZ and DG [133, 134].

Crosstalk between mitochondrial energy homeostasis and Wnt signaling is important in neural crest migration, specification, and in the formation of tissues via transcription factor FoxD3 regulation, as demonstrated via in vivo zebrafish OXPHOS-defective models. FoxD3 ensures correct embryonic development and contributes to the maintenance of cell stemness, as well as to the induction of epithelial-to-mesenchymal transition [127]. Wnts are also expressed abundantly in close vicinity of DAergic neurons during embryonic development and regulate genetic networks that are required to establish progenitor cells and terminal differentiation of DAergic neurons in the later stage of embryogenesis. This has been confirmed in *Wnt-1* knockout mice showing reduced expression of Lmx1A, Ngn2, and Mash1, and consequent progenitor cell proliferation impairment and subsequent differentiation into postmitotic DAergic neurons [125].

In adult niches, the canonical Wnt pathway activates cell proliferation through expression of the transcription factor neurogenic differentiation 1 (NeuroD1) and the activation of long interspersed nuclear elements 1 (LINE1) [135]. Wnt proteins also promote neuronal differentiation and maturation through β-catenin/neurogenin 2 signaling in the DG and β-catenin/homeodomain interacting protein kinase 1 (Hipk1) in the SVZ [136, 137]. The reduction of neurogenesis observed with aging is influenced by inhibitors expressed through the Wnt pathway such as dickkopf WNT signaling pathway Inhibitor 1 (Dkk1) and secreted frizzled-related protein 3 (sFRP3) in the hippocampus, and p-53 induced phosphatase 1 (Wip1) and dickkopf WNT signaling pathway Inhibitor 3 (Dkk3) in the SVZ [138]. A balance of the Wnt/β-catenin signaling is required to support long-term neurogenesis in the adult brain, while non-canonical Wnt through the JNK pathway is required for neuronal differentiation and synapse modulation [136]. Additionally, both canonical and non-canonical Wnt ligands such as Wnt3a and Wnt5a lead to an increase of the β-catenin level together with NPC proliferation and differentiation[128, 139]. Astrocytes in adult niches, together with bone morphogenic proteins 2 and 4 (BMP2/4), promote NSC proliferation by producing Wnt3a/Wnt7a ligands [140, 141]. On the other hand, Wnt/β-catenin signaling negatively affects oligodendrogenesis while GSK3 inhibition promotes myelin basic protein (MBP) expression and oligodendrocyte maturation [142–145]. Altogether, impairments of the canonical and non-canonical Wnt pathways lead to psychiatric, neurodegenerative, and neurodevelopmental disorders (autism spectrum disorders, intellectual disability, micro and macrocephaly, Alzheimer's Disease, Parkinson's Disease, schizophrenia, etc.) [118].

### Mitochondrial diversity in cellular subtypes

In the adult brain, distinct mitochondrial metabolic traits have been observed between all cellular types of the nervous system, including at inter-neuronal and intra-neuronal levels [146]. Synaptic mitochondria express less OXPHOS subunits and proteins involved in calcium buffering than non-synaptic mitochondria [147].

Moreover, mtDNA content varies between neurons of different sub-regions of the hippocampus, with CA2 and CA3 neurons having an higher amount of mtDNA per cell in comparison with CA1 and DG neurons [148, 149]. In addition, mitochondria trafficking could vary among different cell types, and it was demonstrated that mitochondria in both astrocytes and myelin sheets show lower movements and smaller cristae than in neurons [150, 151]. It is well established that neurons are highly oxidative, while astrocytes and oligodendrocytes mainly rely on glycolysis for energy production and are less sensitive to OXPHOS alteration than neurons, whereas microglia can shift between the two metabolisms depending on external stimuli [152–154]. Furthermore, a recent in vivo study revealed how metabotropic glutamate receptor 5 (mGluR5) monitors glutamatergic synaptic activity in astrocytes by modulating the mitochondrial biogenesis through PGC-1α, thus adapting energy demands of the first weeks of life to further support excitatory synaptogenesis [155].

### Mitochondrial dysfunction: implication in neurodevelopmental and neurodegenerative disorders

Mitochondrial integrity and function are crucial for the proliferation, differentiation, and maintenance of NSCs during neural development. Genetic and epigenetic modifications of proteins and metabolites of mitochondrial pathways guiding neurogenesis can cause mitochondrial dysfunction and consequently lead to childhood neurodevelopmental disorders, with clinical presentations ranging from syndromic autism, intellectual disability, and epileptic encephalopathies to childhood-onset neurodegeneration [156].

NPCs are dependent on OXPHOS function being the shift from glycolysis to oxidative mitochondrial metabolism essential for differentiation. Genetically inherited defects in genes encoding protein subunits of OXPHOS complexes may compromise OXPHOS function since the early stages, thus consequently causing early onset of Leigh syndrome, leukoencephalopathy, or other mitochondrial encephalopathies.

Neural cultures derived from iPSCs of patients carrying variants in nuclear genes encoding Complex I subunit (NDUFS4) or complex IV assembly factor (SURF1) have demonstrated that NPCs were unable to shift toward OXPHOS and retained proliferative glycolytic state. Although a minimal residual OXPHOS activity was still present and glycolysis increased to compensate for the OXPHOS defect, neurons remained in an immature state and underwent exhaustion, with a consequent failure to instruct morphogenesis and impairment of neural generation. Brain organoids (3D cultures) generated from the same neural culture further demonstrated the inability to reproduce a physiological cytoarchitecture of neural layers and showed a reduction of organoid size [157]. The immaturity of neurons and cytoarchitecture disorganization also affect synaptogenesis and neural circuits and are responsible for early-onset cognitive defect, delayed acquisition in motor milestones, neurological pyramidal or extra-pyramidal signs, and seizures in patients with Leigh or Leigh-like syndrome (#256,000). Patients also present a typical brain MRI pattern with increased T2 signals in basal ganglia and brainstem, high-energy demand regions that are particularly vulnerable to OXPHOS defects.

**Fig. 2:**
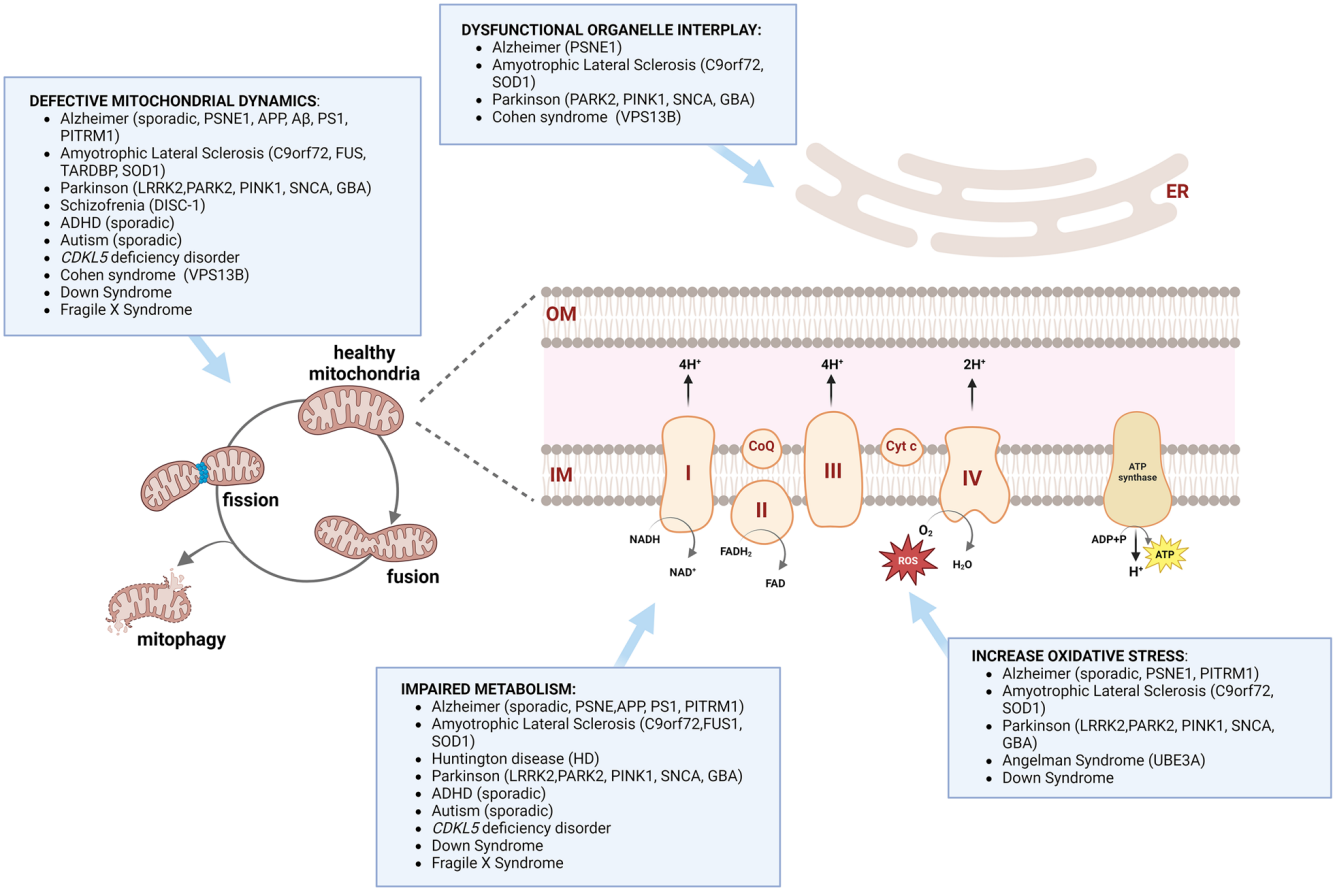
Mitochondrial dysfunction in dynamics, metabolism, oxidative and stress and organelle interplay in neurodevelopmental and neurodegenerative disorders (Created with Biorender)

In addition to primary mitochondrial disorders, mitochondria dysfunction may play a role in the disease mechanism of intellectual disability due to defective proteins not primarily linked to mitochondria [156]. In the same way, impairment in relevant pathways previously mentioned for neuronal proliferation and commitment could have a detrimental effect on the central nervous system, thus leading to neurodevelopmental and neurodegenerative disorders (Fig. 2).

As an example, a neuron-specific proximity-labeling proteomics (BioID2) study was exploited to identify a link between Autism spectrum disorders (ASD) risk genes and convergent pathways. The protein–protein interaction network map unveiled that 41 ASD risk genes are needed for proper cellular respiration, with the mitochondrial/metabolic processes and Wnt signaling pathways as the most relevant pathways shared between those genes [158].

In accordance with data that suggest how mitochondrial dysfunction could be a common denominator for neurodevelopmental disorders, another example is represented by Fragile X syndrome (#300,624), a neurodevelopmental disorder characterized by moderate to severe mental retardation, macroorchidism, and distinct facial features, caused in the majority of cases by an unstable expansion of a CGG repeat in the FMR1 gene and abnormal methylation, which results in suppression of FMR1 transcription and decreased protein levels in the brain [159]. Recent studies in vivo and in vitro models for Fragile X syndrome have demonstrated that the FMRP protein regulates the closure of the ATP-synthase c-subunit leak. Neural and astrocyte cells from *Fmr1*^−/y^ mice had inefficient respiration and an abnormal “proton leak”, which led to ineffective oxidative phosphorylation [156]. Single-cell transcriptome analysis in the hiPSC model of FXS investigated neuronal GABA functional switch, a key event of fetal development that converts GABA from being an excitatory neurotransmitter to an inhibitory one. Analysis at two stages of GABAergic neurogenesis showed similar behavior in FXS and control hiPSC at the earlier time point, while later-stage FXS cells revealed an aberrant neurogenic origin of the delayed GABA hyperpolarizing action related to decreased mitochondria function. Gene set enrichment analysis at later-stage identified the most relevant upregulated gene ontology processes as those involved in cytoplasmic translation, neuroblast proliferation, and Wnt signaling, while genes related to more mature neuronal processes as synaptic and mitochondrial functions resulted in the most down-regulated. Consequently, FXS GABAergic progenitors are arrested at a younger state and the unceasing generation of immature inhibitory neurons leads to dysfunctional neural networks [160]. Likewise, mitochondrial dysfunctions were reported also in a mouse model of *Cdkl5* deficiency disorder (#312,750), an atypical form of Rett syndrome characterized by early-onset epilepsy. Magnetic resonance spectroscopy in the hippocampus of *Cdkl5* KO mice highlighted a reduction of N-acetyl aspartate peak connected to decreased ATP levels and diminished activation of AMPK, a known inhibitor of mTOR [161, 162].

Damaged mitochondria with increased levels of oxidative stress were also observed in fibroblast patients with Down syndrome (DS, HSA21), the most common genetic disorder caused by trisomy of chromosome 21. Patients’ fibroblasts were characterized by an aberrant mitophagy and hyper-activation of mTOR, followed by imbalances in autophagy flux regulation and detrimental effects on mitochondrial turnover. Nevertheless, a compound able to inhibit mTORC1 and mTORC2 (AZD8055) restored the abnormal accumulation of damaged mitochondria and opened the possibility of targeting mTORC1-mTORC2 inhibition as a candidate therapeutic strategy for Down Syndrome[163].

Also, *Disrupted-in-Schizofrenia 1* (*DISC1*) is a gene involved in adult and embryonic neurogenesis, and it is associated with a risk of developing mental illness as well as with impaired mitochondrial trafficking within neuronal axons [164, 165]. Shrikanth et al. generated DISC1 disruption in hiPSCs by using TALENS, obtaining the loss of DISC1 long isoforms, reduction of fate marker as FOXG1 and TBR2 progenitor cells, aberrant WNT signaling and impaired transcriptional profile of NPCs and neurons [166]. The same group developed cerebral organoids generated from mutant DISC1-ex8^wt/γ^ hiPSCs, which showed important morphological changes, with disorganized and small rosettes, induced by abnormal activity of the WNT pathway as well as reduction of the BRN2 transcription factor, involved in TBR2^+^ NSCs generation and migration [167].

Mitochondrial dysfunction in stem cell models derived from patients with neurodevelopmental disorders are summarized in Table 1: References are listed in Additional file 1.

**Table 1:** Neurodevelopmental disorders: mitochondrial dysfunction in patients’ derived stem cell models

Neurodevelopmental disorders	Type of cells	Oxidative stress	Mitochondrial dynamics	Oxphos metabolism	Organelle interplay	References
Angelman Syndrome (UBE3A)	Neuronal progenitor cells	Increased ROS and oxidative stress	–	–	–	Simchi et al. 2023
Sporadic ADHD	Dopaminergic neurons	–	Reduced mitochondrial content and trafficking	Reduced ATP level	–	Nguyen et al. 2019
Sporadic Autism	Dopaminergic neurons	–	Reduced mitochondrial content	Reduced ATP level	–	Nguyen et al. 2018
*CDKL5* deficiency disorder	Cortical neurons	–	Reduced mitochondrial trafficking velocity and increased stationary mitochondria	Reduction of expression of NDUFB8, SDHB, and COXII. Reduction in Complex I, II, and IV activities. Increased Citrate Synthase activity	–	Van Bergen et al. 2021
Cohen Syndrome (VPS13B)	Cortical neurons	–	Aberrant mitochondrial morphology	–	Increased ER-mitochondria contact sites	Shnaider et al. 2023
Down Syndrome	Dopaminergic neurons	Increased ROS	Reduced mitochondrial content	Reduced ATP level	–	Sun et al. 2021
Down Syndrome	GABAergic interneurons	–	Abnormal mitochondrial aggregation	Reduced level of basal respiration, ATP production, maximum respiration, and spare respiration capacity	–	Xu et al. 2022
Down Syndrome	Medial ganglionic eminence organoid	–	Abnormal mitochondrial aggregation	–	–	Xu et al. 2022
Fragile X Syndrome (FMRP)	Cortical neurons	–	Reduced mitochondrial content and aberrant morphology Reduced expression of fusion and mitochondrial biogenesis proteins and increased fission proteins	Reduced ATP level	–	Shen et al. 2023

– Not reported; ATP: adenosine triphosphate; ER: endoplasmic reticulum; iPSC: induced pluripotent stem cell; mtDNA: mitochondrial DNA; OXPHOS: oxidative phosphorylation; ROS: reactive oxygen species. References are listed in Additional file 1

When early neurogenesis is completed, mitochondria are responsible for the maintenance of the metabolic state of mature neurons and guide the adult neurogenesis in niches located in the subgranular and subventricular zones. Primary mitochondrial dysfunction or metabolic requirements that go beyond a sustainable level of metabolic flexibility challenge cellular resilience and brain function and contribute to neurodegeneration [168]. Mitochondrial dysfunction in stem cell models derived from patients with neurodegenerative disorders are summarized in Table 2: References listed in Additional file 1.

**Table 2:** Neurodegenerative disease: mitochondrial dysfunction in patients’ derived stem cell models

Neurodegenerative disease	Cells type	Oxidative stress	Mitochondrial dynamics	Oxphos metabolism	Organelle interplay	References
Alzheimer disease						
Sporadic	iPSC	Increased oxidative stress	–	–	–	Hossini et al. 2015
Sporadic	Cortical neurons	–	Impaired mitophagy, increased mitochondrial fragmentation	Reduced ATP level	–	Fang et al. [176]
Sporadic	Cortical neurons	Increased ROS and oxidative stress with mtDNA damage	Normal level of dynamics proteins	Increased protein expression of NDUFB8, UQCRC2, MTCO1, ATP5A	–	Birnbaum et al. 2018
PSEN1	Cortical neurons	–	Aberrant mitochondrial network size, morphology, and trafficking Dynamics proteins deregulation	Lower expression levels of cytochrome c oxidase (complex IV), cytochrome c reductase (complex III), succinate dehydrogenase (complex II), NADH: CoQ reductase (complex I), and ATP synthase (complex V)	Impaired lysosomal function with consequent accumulation of damaged mitochondria	Martin-Maestro et al. [174]; Martin-Maestro et al. 2017; Li et al. 2020
PSEN1	Cholinergic neurons	Increased ROS and oxidative stress with mtDNA damage	–	Reduced ATP level	–	Oka et al. 2016
PSEN1	Astrocytes	Increased ROS	–	–	–	Oksanen et al. 2017
APP	Cortical Neuron	–	Impaired mitophagy, increased mitochondrial fragmentation	Reduced ATP level	–	Fang et al. [176]
Amyotrophic lateral sclerosis						
C9orf72	Motor neurons	Increased ROS and oxidative stress with mtDNA damage	Aberrant mitochondrial morphology	Reduced basal and maximal respiration	ER stress	Lopez-Gonzalez, 2016; Mehta et al. 2021; Dafinca et al. 2016
FUS	Motor neurons	–	Aberrant mitochondrial trafficking	ER-mitochondrial overlay	–	Guo et al. 2018
TARDBP	Motor neurons	–	Reduced mitochondrial motility	–	–	Fazal et al. 2021
SOD1	Motor neurons	Increased oxidative stress	Aberrant mitochondrial morphology and trafficking	Reduced ATP level	ER stress	Günther et al. 2022; Kiskinis et al. 2014
Huntington disease						
HD	Cortical neurons	–	–	Reduce maximal respiratory capacity	–	An et al. 2012
Parkinson disease						
LRRK2	iPSC	Increased oxidative stress	–	–	–	Nguyen et al. 2011
LRRK2	Dopaminergic neurons	Increased oxidative stress and mtDNA damage	Reduced mitochondrial content and trafficking. Drp1-mediated dysfunction with mitochondrial fragmentation	Reduced maximal respiratory capacity and ATP/ADP level. Undetectable protein expression of NDUFB8 and COX II, decreased levels of UQCRC2	–	Schwab et al. 2017; Nguyen et al. 2011; Sanders et al. 2014; Su et al. 2013
PARK2	Neurons	Increased oxidative stress	Abnormal mitochondrial morphology	–	Aberrant tubulovesicular organelle adjacent to Golgi	Imaizumi et al,, [185]
PARK2	Neuropeptidergic Neurons	–	–	–	Increased ER-Mitochondria contact sites	Valadas et al. 2018
PARK2	Dopaminergic Neurons	Increased ROS	Abnormal mitochondrial content and morphology	Reduced Complex I	Increased ER-Mitochondria contact sites	McLelland et al. 2017; Chung et al. 2016; Zanon et al. 2017; Shaltouki et al. 2015
PINK1	iPSC	Increased oxidative stress	–	–	–	Nguyen et al. 2011; Cooper et al. 2012
PINK1	Neuropeptidergic Neurons	–	–	–	Increased ER-Mitochondria contact sites	Valadas et al. 2018
PINK1	Dopaminergic neurons	Increased ROS	Abnormal mitochondrial morphology	Reduced level of ATP	–	Chung et al. 2016; Vos et al. 2017
SNCA	Dopaminergic neurons	Increased oxidative stress	Abnormal mitochondrial morphology and trafficking. Reduction of DRP1expression	Reduced maximal respiratory capacity and ATP production	ER stress; reduced ER-mitochondria contact sites	Zambon et al. 2019; Little et al. 2018; Paillusson et al. 2017; Prots et al. 2018
GBA	Dopaminergic neurons	Increased oxidative stress	Abnormal mitochondrial morphology. Reduction of DRP1, OPA1 and MFN1 expression	Reduced maximal respiratory capacity and ATP production. Reduced Complex I activity	ER stress Reduction of mitochondrial-lysosomal co-localization	Schöndorf et al. 2018

–: Not reported; ATP adenosine triphosphate; ER: endoplasmic reticulum; iPSC: induced pluripotent stem cell; mtDNA: mitochondrial DNA; OXPHOS: oxidative phosphorylation; ROS: reactive oxygen species. References are listed in Additional file 1

Alzheimer's disease (AD) is one of the most common neurodegenerative disorders. The diagnosis is clinical and biological, requiring the presence of a clinical phenotype characterized by amnestic syndrome of the hippocampal type, the posterior cortical atrophy variant and the logopenic variant primary progressive aphasia and the biological hallmarks of extracellular β-amyloid plaques and intracellular hyperphosphorylated tau tangles [169, 170]. Multiple mitochondrial mechanisms may contribute to sporadic AD development, including reduced glucose and oxygen metabolism, altered mitochondrial morphology, defective cytochrome oxidase function, and reduction in mtDNA content [171]. Among these mechanisms, a connection between Aβ and the balance between mitochondria fusion and fission has been observed in AD patients and in murine primary hippocampal neurons [172]. In Familial Alzheimer’s disease due to defective presenyl-1 (PS1) impairment in the transcriptional activation of Wnt-dependent genes has been found, thus affecting numerous cellular structures and mechanisms involved in cell fate such as microtubules, neurofilaments, and neural adhesion [173]. PS1^AZ46E^ and PS1^M146L^ neurons from AD patients revealed that mutations in this gene cause impaired mitophagy and metabolic changes due to a reduced expression of OXPHOS enzymes [174, 175]. Similarly, patient-derived APP^V717L^ cortical neurons showed a reduction of the TBL1 and ULK1 proteins, increased mitochondrial fragmentation, accumulation of defective mitochondria, and reduced energy metabolism [176]. Impaired activity of pitrilysin metallopeptidase 1 (PITRM1) or presequence protease (PreP), a mitochondrial matrix enzyme implicated in the protein import process by digesting the mitochondrial targeting sequence (MTS), is deemed to be involved in the development of Alzheimer’s Disease [177]. Cerebral organoids generated from CRISPR/Cas9 generated PITRM1-KO hiPSCs showed neuronal cell loss, signs of the mitochondrial stress response, and mitochondrial clearance accompanied by AD-like accumulation of APP and Aβ and increment in mitochondrial unfolded protein response (UPR^mt^) induction. Interestingly, scRNA data revealed that common mitochondrial pathways such as the OXPHOS and sirtuin pathways were dysregulated in neurons, astrocytes, and microglia while the synaptogenesis pathway and inflammatory pathway were specifically dysregulated in neurons and astrocytes, respectively [178]. Moreover, AD patients showed decreased levels of Wnt2b, a member of the Wnt family and a mitochondria‐expressed protein that shuttles between mitochondria and the nucleus, which might influence neuronal damage by acting on Wnt signaling and mitochondrial activity and seemed to correlate with declined cognitive function in vitro and in vivo AD. However, the defect could be restored by recovering the ATP level [179]. Eventually, it was recently demonstrated the role of SOD1, a metalloenzyme that catalyzes the removal of superoxide free radicals, to connect extracellular signals and mitochondrial functioning by permitting nutrients to maintain OXPHOS functions. The activation of lysosomal mTORC1 regulates mitochondrial respiration under the control of mTORC1-catalyzed phosphorylation of SOD1 at T40 residue and the pharmacological inhibition of SOD1 activity impeded respiration in cells and in the mouse cortex of AD. These results underline the importance of the lysosome-to-mitochondria signaling pathway and how its disruption plays a role in developing AD and tuberous sclerosis [180].

Degeneration of DAergic neurons in substantia nigra pars compacta and striatum regions cause Parkinson’s disease (PD), clinically characterized by motor deficits such as bradykinesia, resting tremor, rigidity, and postural instability [181]. Affected neurons show intracellular deposition of Lewy bodies mostly formed by alpha-synuclein (α-syn) [182]. Studies in rodents and biochemical and histological analysis of patient brain tissues demonstrated mitochondrial Complex I deficit as well as mitophagy and mtDNA alterations, suggesting the role of mitochondrial dysfunction in the disease pathogenesis and a potential link between mutations in α-syn and impairments in mitochondrial dynamics [183, 184]. In a PD in vitro model, iPSC-derived mutant PARK2 neurons exhibited increased oxidative stress and aberrant activation of the NRF2 pathway with impaired mitochondrial function and α-synuclein accumulation [185]. iPSC-derived mutant PARK2 dopaminergic neurons, carrying a GFP expression cassette at the TH locus, showed small and less functional neurons with a decline in mitochondrial membrane potential, suggesting a mitochondria-dependent mechanism involved in dopaminergic neuron susceptibility to PD [186]. In *Drosophila melanogaster* mutations in PINK1 caused degenerations in DA neurons due to mitochondrial dysfunctions and ROS increase together with reduced MnSOD protein expression. Overexpression of the Wnt2 gene rescued those phenotypes in PINK1B9 transgenic Drosophila, and augmented the mitochondrial biosynthesis-related gene PGC-1α levels, although without increasing the protein expression of β-catenin, suggesting a protective effect of Wnt2 gene in PD PINK1B9 transgenic Drosophila via non-canonical pathway [187]. Additionally, activation of Wnt/β-catenin signaling improves behavioral functions and protects the nigral DAergic neurons by increasing mitochondrial functionality in Parkinsonian rats. Specifically, Wnt/β-catenin reduces apoptotic signaling, autophagy, and ROS generation, significantly increases fusion-related factors Mfn-2, OPA-1, PGC-1α, and TFAM, and improves mitochondrial membrane potential which promotes mitochondrial biogenesis [125].

Mitochondrial dysfunction has been also implicated in the pathogenetic mechanism in other inherited or sporadic neurodegenerative disorders such as Frontotemporal dementia, Amyotrophic lateral sclerosis, and Huntington's disease and is considered a potential target for preventing disease progression (Table 2, Additional file 1) [188].

## Conclusions

Mitochondria play a central role in neuronal cell fate by regulating their metabolic state and directing differentiation, commitment, and maintenance. Mitochondrial shape remodeling with a metabolic shift towards neurogenesis is the first step, while epigenetic and metabolic signals interplay acts downstream on several proteins, with HIF-related pathways acting as a central sensor. Additional studies are required to better characterize the epigenetic control of neurogenesis and identify potential targets for therapy with either genetic or pharmacological treatment. Genetic defects in proteins involved in the mitochondrial control of neurogenesis or secondary dysfunction in mitochondrial metabolism and dynamics are responsible for neurodegenerative and neurodevelopmental disorders. In our review, we have provided a comprehensive analysis of the mitochondrial dynamics, energy metabolism, oxidative stress, and organelles interplay in stem cell models derived from patients with neurodevelopmental and neurodegenerative disorders. With specific regard to neurodegenerative disorders, data from stem cell models have corroborated previous observations in human post-mortem tissue and in vivo disease models. Therefore, those models will be fundamental for further clarifying the metabolic pathways involved in neurogenesis and identifying therapeutic targets for neurodevelopmental and neurodegenerative disorders. They have been already successful in identifying pharmacological treatment with a repurposing drug approach and they will be a reliable and feasible model for evaluating efficacy and safety of novel experimental therapies.

In conclusion, mitochondrial dysfunction has a central role in neurodegenerative and neurodevelopmental disorders. Future studies are needed to identify genetic or pharmacological approaches able to ameliorate the clinical and biological phenotype of these disorders.

### Supplementary Information

**Additional file 1.** Supplementary references.

## Abbreviations

NSC: Neural stem cell
mtDNA: Mitochondrial DNA
OXPHOS: Oxidative phosphorylation
ROS: Reactive oxygen species
TCA: Tricarboxylic Acid
ETC: Electron Transport Chain
IMM: Inner mitochondrial membrane
OMM: Outer mitochondrial membrane
NPCs: Neural progenitor cells
NE: Neuroepithelial
RG: Radial glial
CNS: Central nervous system
GFAP: Glial fibrillary acidic protein
VZ: Ventricular zone
BP: Basal progenitors
IP: Intermediate progenitor
SVZ: Subventricular zone
SGZ: Subgranular zone
OB: Olfactory bulb
RMS: Rostral migratory stream
PK: Pyruvate kinase
aKG: A-ketoglutarate
SAM: S-adenosyl methionine
HIF-1α: Hypoxia-Inducible-Factor HIF-1α
COX: Cytochrome C oxidase
MAM: Mitochondria-associated ER membrane
TSC: Tuberous sclerosis complex
PCP: Planar cell polarity
ASD: Autism spectrum disorders
DS: Down syndrome
AD: Alzheimer disease
PD: Parkinson’s disease
