# Supplementary material for: Mitochondrial metabolism in neural stem cells and implications for neurodevelopmental and neurodegenerative diseases
Source: J Transl Med. 2024 Mar 4;22:238. doi: 10.1186/s12967-024-05041-w (PMC10910780; doi:10.1186/s12967-024-05041-w)
Supplement: Supplementary file 1 — Additional file 1. Supplementary references. [file 12967_2024_5041_MOESM1_ESM.docx]

**Supplementary references**

**An et al, 2012** doi.10.1016/j.stem.2012.04.026.

**Birnbaum et al, 2018** doi.org/10.1016/j.scr.2018.01.019

**Chung et al, 2016** doi.org/10.1016/j.stemcr.2016.08.012

**Dafinca et al, 2016** doi.org/10.1016/j.stemcr.2020.03.023

**Fang et al, 2019** doi.org/10.1038/s41593-018-0332-9

**Fazal et al, 2021** doi 10.15252/embj.2020106177

**Günther et al, 2022** doi.org/10.3390/cells11071246

**Guo et al, 2018** doi. 10.1038/s41467-017-00911-y

**Hossini et al, 2015** doi. 10.1186/s12864-015-1262-5

**Imaizumi et, 2012** doi. 10.1186/1756-6606-5-35.

**Kiskinis et al, 2014** doi.org/10.1016/j.stem.2014.03.004

**Li et al 2020** doi.org/10.1111/cpr.12798

**Little et al, 2018** doi.10.1038/s41598-018-27058-0

**Lopez-Gonzalez, 2016** doi.org/10.1016/j.neuron.2016.09.015

**Martin-Maestro et al, 2019** doi.org/10.1007/s12035-019-01665-y

**Martin-Maestro et al, 2017** doi: 10.3389/fnmol.2017.00291

**Mehta et al, 2021** doi.org/10.1007/s00401-020-02252-5

**Nguyen et al, 2018** doi.org/10.1016/j.bbrep.2018.09.004

**Nguyen et al, 201**9 doi.org/10.1016/j.bbrc.2019.04.084

**Nguyen et al, 2011** doi.10.1016/j.stem.2011.01.013

**Oka et al, 2016** doi.10.1038/srep37889

**Oksanen et al, 2017** doi.org/10.1016/j.stemcr.2017.10.016

**Paillusson et al, 2017** doi.10.1007/s00401-017-1704-z)

**Prots et al, 2018** doi.10.1073/pnas.1713129115

**Sanders et al, 2014** doi.org/10.1016/j.nbd.2013.10.013

**Schöndorf et al, 2018** doi.org/10.1016/j.celrep.2018.05.009

**Schwab et al, 2017** doi.org/10.1016/j.stemcr.2017.10.010

**Shen et al, 2023** doi.org/10.1016/j.neuron.2023.09.014

**Shnaider et al, 2023** doi.org/10.3390/cells12232702

**Simchi et al, 2023** doi.org/10.1038/s41380-023-02038-7

**Su et al, 2013** doi.10.1093/hmg/ddt301

**Sun et al, 2021** doi. 10.1096/fba.2021-00086

**Valadas et al, 2018** doi.org/10.1016/j.neuron.2018.05.022

**Van Bergen et al, 2021** doi.org/10.1016/j.nbd.2021.105370

**Vos et al, 2017** doi.org/10.1083/jcb.201511044

**Xu et al, 2022** doi.org/10.1016/j.bbadis.2022.166388

**Zambon et al, 2019** doi.10.1093/hmg/ddz038
